# Effectiveness and safety of self-pulling and latter transected Roux-en-Y reconstruction in totally laparoscopic distal gastrectomy

**DOI:** 10.3389/fonc.2022.916692

**Published:** 2022-10-07

**Authors:** Defei Chen, Fuyu Yang, Saed Woraikat, Chenglin Tang, Kun Qian

**Affiliations:** Department of Gastrointestinal Surgery, The First Affiliated Hospital of Chongqing Medical University, Chongqing, China

**Keywords:** totally laparoscopic distal gastrectomy, Roux-en-Y reconstruction, gastric cancer, self-pulling and latter transection, laparoscopy-assisted distal gastrectomy

## Abstract

**Background:**

Self-pulling and latter transection (SPLT) reconstruction has been applied in total laparoscopic total gastrectomy and BI reconstruction (known as Delta SPLT) in total laparoscopic distal gastrectomy (TLDG) in some previous studies. This approach can reduce the technical difficulty of the surgery as well as the quantity of cartridges required, with manageable safety. Here, we used SPLT to complete Roux-en-Y reconstruction in TLDG and evaluated the safety and effectiveness of this novel method by comparing it with conventional Roux-en-Y reconstruction in laparoscopy-assisted distal gastrectomy (LADG).

**Methods:**

Patients with gastric cancer who underwent SPLT-TLDG or LADG between June 2019 and September 2021 were retrospectively analyzed. Baseline information and postoperative short-term surgical outcomes of the two groups were compared.

**Results:**

A total of 114 patients with gastric cancer were included in the study. Patients underwent SPLT-TLDG (n = 73, 64.0%) or LADG (n = 41, 36.0%). No patient underwent open surgery. There were no differences in patient demographics or tumor characteristics between the two groups. The mean intraoperative blood loss was 47.1 ± 34.3 ml in the SPLT-TLDG group, which was significantly less than that in the LADG group (P = 0.022). There were no significant differences in operation time, harvested lymph nodes, time to first flatus, time to liquid intake, or postoperative hospital stay between the two groups. Nine and five patients had short-term postoperative complications in the SPLT-TLDG and LADG groups, respectively.

**Conclusion:**

We introduced a self-pulling and latter transected Roux-en-Y reconstruction (SPLT-RY) for use in TLDG. We showed that SPLT-RY reconstruction in TLDG is a safe and feasible surgical method in terms of short-term surgical outcomes and has the advantages of simplifying the reconstruction.

## Introduction

Laparoscopy-assisted gastrectomy (LAG) for gastric cancer was first reported in 1994 (1). LAG is less invasive, and patients recover earlier than with open gastrectomy (2–4). Total laparoscopic gastrectomy (TLG) was first reported with intracorporeal Billroth II (BII) reconstruction using laparoscopic linear staplers (5). TLG was proven to be reliable and feasible in patients with gastric cancer (6–8). Nevertheless, for surgeons, TLG remains a surgical challenge due to the difficulty of intracorporeal reconstruction (9).

The reconstruction method for total laparoscopic distal gastrectomy (TLDG) includes Billroth I (BI) reconstruction (10), BII reconstruction (11), Roux-en-Y (RY) reconstruction (12), and uncut RY reconstruction (13). BI reconstruction has specific requirements in terms of gastric cancer location. BII and RY reconstruction both had wide indications, but RY seemed to be a preferred reconstruction after TLDG in terms of short- and long-term surgical outcomes (14, 15); however, RY is a complex procedure and is markedly more expensive and requires more surgical skills than do BI and BII. Self-pulling and latter transection (SPLT) reconstruction, which can reduce the technical difficulty of the surgery and the quantity of cartridges required, with manageable safety, has been used in total laparoscopic total gastrectomy and with BI reconstruction (known as Delta SPLT) in TLDG in some previous studies (16, 17).

To simplify RY reconstruction, we implemented SPLT to complete RY reconstruction in TLDG. The purpose of this study was to describe the SPLT-RY procedure in TLDG and to evaluate its effectiveness and safety by comparing it with conventional RY reconstruction in laparoscopy-assisted distal gastrectomy (LADG).

## Patients and methods

### Patients

The patient selection criteria were as follows. Inclusion criteria: Patients had undergone SPLT-TLDG or LADG between June 2019 and September 2021; the procedures were performed by the same surgeon, who had more than 10 years of surgical experience; gastric adenocarcinoma was confirmed by pathological biopsy; the tumor was located in the gastric antrum, lesser curvature of the stomach, or corner of the stomach; preoperative CT suggested T1–3, without detection of any distal metastasis (M0); and patients were informed of the advantages and disadvantages of the two procedures before the operation and chose a surgical method by signing an informed consent form. Exclusion criteria: Patients had a serious dysfunction in the heart, lung, bone marrow, kidney, or liver; patients had other synchronous malignancies; or patients had undergone combined resection of other organs.

### Procedures of SPLT-TLDG

After general anesthesia, the patient was placed in a split-leg position. The surgeon was positioned on the right side, the assistant was positioned on the left side of the patient, and the cameraman stood between the legs of the patient.

**Figure 1:**
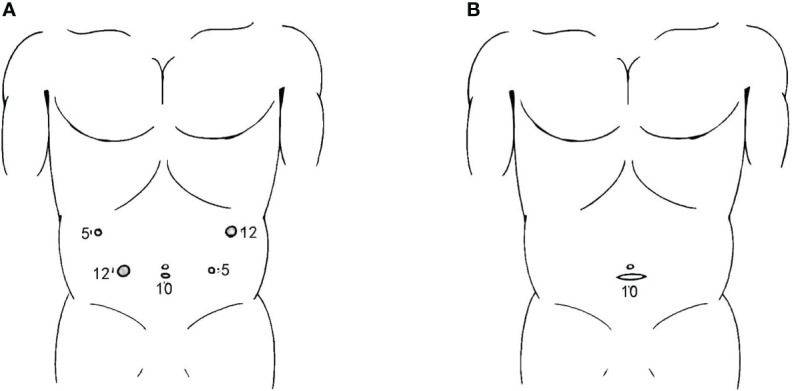
Incisions for SPLT-RY in TLDG. **(A)** Placement of the trocars; **(B)** abdominal transverse incision used to remove the resected specimen. SPLT-RY, self-pulling and latter transected Roux-en-Y; TLDG, total laparoscopic distal gastrectomy.

The port placement process started after establishing pneumoperitoneum, which maintained a pressure of 1.6 kPa. Three trocars were inserted for laparoscopic exploration, and after confirming that there was no metastasis, another two trocars were inserted. The positions of the five trocars were as follows: a 10-mm trocar was inserted 1 cm below the umbilicus, 12- and 5-mm trocars were inserted 2 cm below the lower edge of the costal arch, at the left and right anterior axillary lines; and 12-mm and 5-cm trocars were inserted on both sides of the lower quadrant on the umbilicus line (**Figure 1A**).

**Figure 2:**
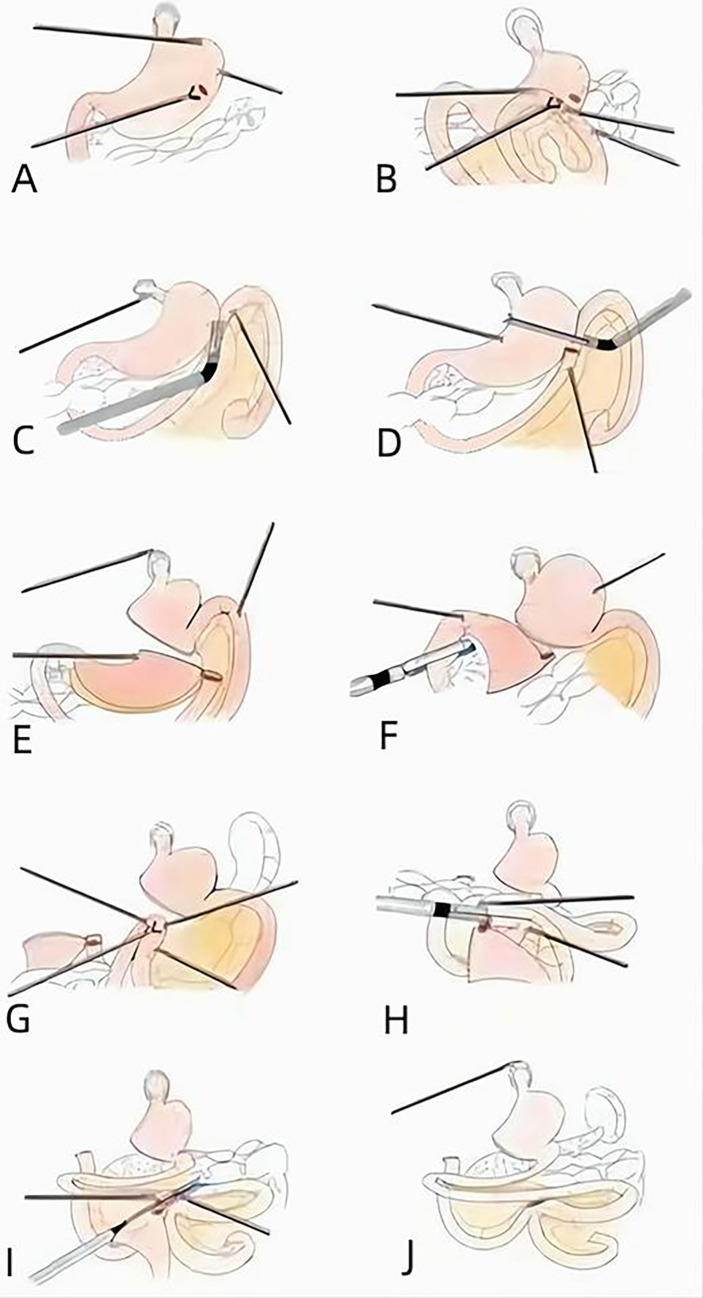
Procedure of SPLT-RY in TLDG. **(A)** Puncturing the posterior wall of the proximal stomach. **(B)** Puncturing the antimesenteric border of the jejunum. **(C)** Anastomosis of the proximal stomach and distal jejunum. **(D)** Closing the common opening. **(E)** Cutting off the proximal stomach. **(F)** Disconnecting the distal stomach and duodenum. **(G)** Puncturing the antimesenteric border of the small intestine. **(H)** Anastomosis of the small intestine and proximal jejunum. **(I)** Closing the common opening. **(J)** Removing the distal stomach. SPLT-RY, self-pulling and latter transected Roux-en-Y; TLDG, total laparoscopic distal gastrectomy.

D2 lymph node (LN) dissection was performed according to the Japanese Gastric Cancer Association guidelines (18). The SPLT-RY reconstruction was then conducted according to the following procedures: We punctured the posterior wall of the proximal stomach, at least 5 cm away from the tumor (**Figure 2A**). We also punctured the antimesenteric border of the jejunum, 15–20 cm away from the Treitz ligament (**Figure 2B**). We then tailored the mesentery along the jejunum. We made an anastomosis of the proximal stomach and distal jejunum by using a linear cutting stapler from the right 12-mm trocar (**Figure 2C**). We closed the common opening of the proximal stomach and distal jejunum and cut off the distal stomach and proximal jejunum by linear cutting stapler from the left 12-mm trocar at the same time and then sutured the opening of the distal stomach (**Figures 2D, E**). Next, we disconnected the distal stomach and duodenum from the right 12-mm trocar (**Figure 2F**). Subsequently, we punctured the antimesenteric border of the small intestine 40 cm away from the proximal stomach and distal jejunum anastomosis (**Figure 2G**). We also punctured the antimesenteric border 1 cm away from the margin of the proximal jejunum and made an anastomosis of the common opening of the small intestine and proximal jejunum by linear cutting stapler from the right 12-mm trocar (**Figure 2H**). The common opening of the proximal jejunum and small intestine was closed by linear closure from the right 12-cm trocar (**Figure 2I**), and the distal stomach was removed (**Figure 2J**).

A 3- to 4-cm transverse abdominal incision was made to remove the specimen after reconstruction was completed (**Figure 1B**). After anastomotic stomas were checked for patency, including bleeding or tension, and bleeding was stopped carefully, the transverse incision was closed, and the operation was completed.

### Procedures of LADG

All procedures before reconstruction in LADG were the same as those in SPLT-TLDG. After D2 LN dissection, the pneumoperitoneum was released. A 7- to 8-cm incision was made at the exact center of the epigastrium and was protected using an incision protector. The distal stomach was resected with a linear cutting stapler and was removed from the previously dissected tissues. Side-to-side gastrojejunostomy and jejunojejunostomy were performed using a linear cutting stapler. All anastomotic stomas and stumps were checked carefully to ensure that there was no visible bleeding or tension. Reinforcing with interrupted sutures was used if necessary. The abdominal incision was closed after placing an indwelling drainage tube. The surgery was then completed.

### Postoperative management

**Figure 3:**
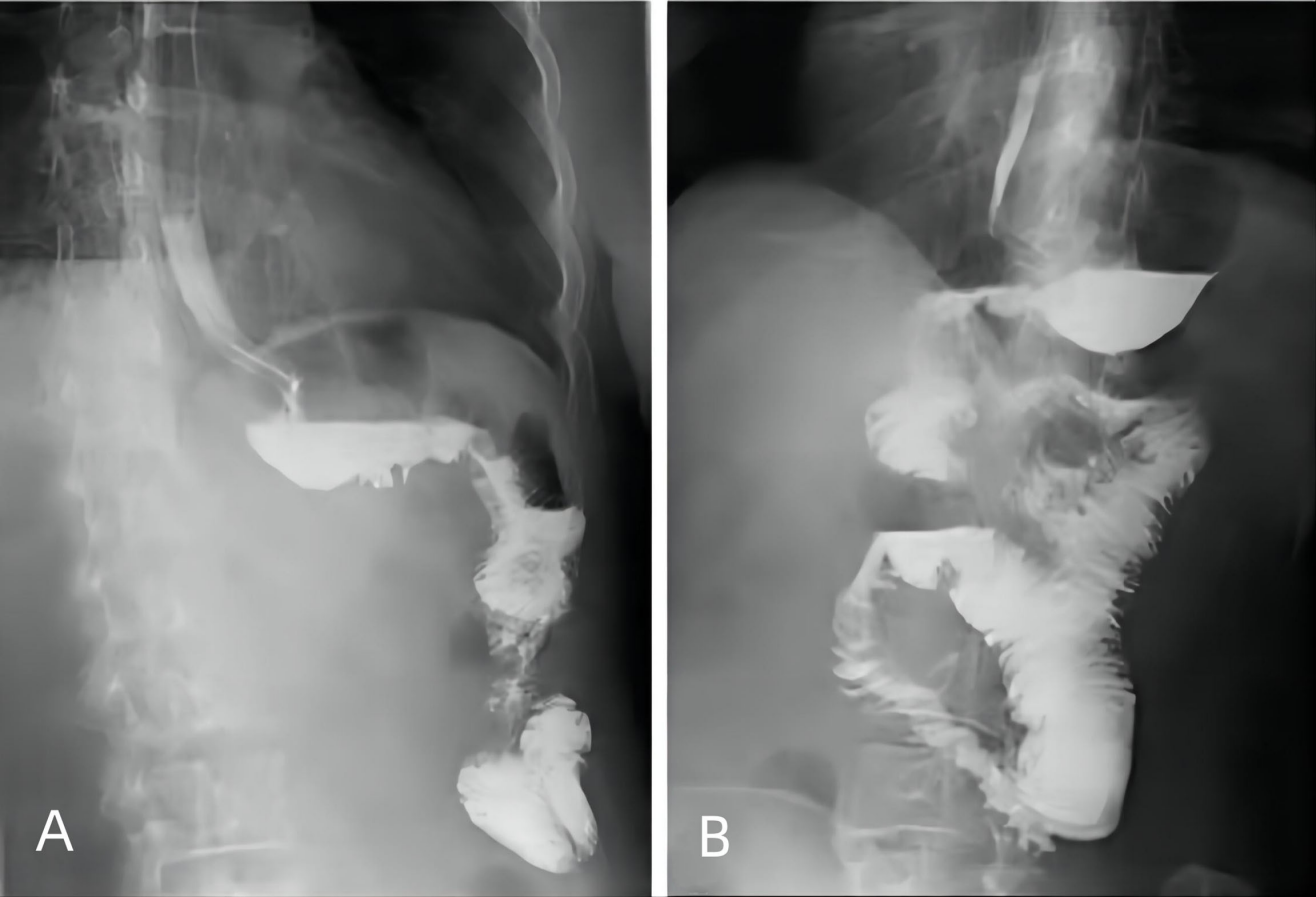
Upper gastrointestinal water-soluble contrast radiography performed 3 days after SPLT-RY in TLDG. **(A)** Anastomosis of the proximal stomach and distal jejunum. **(B)** Anastomosis of the small intestine and proximal jejunum. SPLT-RY, self-pulling and latter transected Roux-en-Y; TLDG, total laparoscopic distal gastrectomy.

All the patients underwent standardized postoperative management. Broad-spectrum antibiotics were used for 48 h during their postoperative hospitalization. Routine octreotide was administered until liquid intake was permitted in both the groups. Upper gastrointestinal water-soluble contrast radiography was typically performed for 3 days after gastrectomy. A liquid diet was recommended if the patient’s flatus recovered or if no anastomosis leakage was found on upper gastrointestinal water-soluble contrast radiography (**Figure 3**). Ambulation was encouraged on the first postoperative day. Patients without complications were discharged once their bowel movements recovered, and they showed no discomfort with the liquid diet.

### Data collection

The baseline information collected from the two groups included sex, body mass index (BMI), preoperative hemoglobin, preoperative albumin, tumor stage, and important history. Intraoperative data collected included operation time, intraoperative blood loss, and harvested lymph nodes. Postoperative data included time to ambulation postoperatively, time to first flatus, time to first fluid intake, postoperative hospital stay, decrease in hemoglobin and albumin levels, and complications.

### Statistical analysis

All statistical analyses were performed using the SPSS software (version 26.0; IBM Inc., Armonk, NY, USA). Differences in continuous variables between the two groups were tested using the Mann–Whitney U test. Differences in ordered categorical variables were compared using chi-square tests. Statistical significance was set at P < 0.05.

## Results

A total of 114 patients were included in this study, and the baseline information of the two groups was compared (**Table 1**). No significant between-group differences were found for sex, BMI, ASA scores, preoperative hemoglobin and albumin levels, tumor characteristics, or medical history, such as abdominal surgery history.

**Table 1:** Patient demographics and tumor characteristics of both groups.

Characteristics	SPLT-TLDG(n = 73)	LADG(n = 41)	P value
Age (years)^a^	61 (35-84)	62 (34-84)	0.545
Sex (male/female) ^b^	41/32	28/13	0.204
BMI (kg/m^2^) ^c^	22.8 ± 2.8	22.5 ± 3.1	0.816
Smoking ^b^	28	23	0.068
Drinking ^b^	22	18	0.139
Abdominal surgery history ^b^	13	9	0.591
Main comorbidity			0.327
Hypertension/T2DM/COPD ^b^	8/6/3	10/1/2	
ASA score (1/2/3/4) ^b^	27/42/4/0	13/24/4/0	0.643
Preoperative hemoglobin (g/L) ^c^	121.9 ± 23.7	116.1 ± 18.4	0.125
Preoperative albumin (g/L) ^c^	39.9 ± 4.2	39.3 ± 4.5	0.116
Neoadjuvant chemotherapy ^b^	18	13	0.417
Tumor size (cm)^c^	3.1 ± 2.0	2.8 ± 1.6	0.535
T stage (T1/T2/T3/T4) ^b^	36/10/22/5	16/9/9/7	0.187
Node stage (N0/N1/N2/N3) ^b^	46/9/9/9	23/4/8/6	0.705
TNM stage (I/II/III/IV) ^b^	38/16/19/0	19/9/13/0	0.789

Data are shown as medians, with ranges in parentheses. ^b^Data are given as n of corresponding groups. ^c^Data are shown as mean ± standard deviation.

T2DM, type 2 diabetes mellitus; COPD, chronic obstructive pulmonary disease; BMI, body mass index; ASA, American Society of Anesthesiologists; SPLT, self-pulling and latter transected; TLDG, total laparoscopic distal gastrectomy; LADG, laparoscopy-assisted distal gastrectomy.

The operative and postoperative data of the study patients are shown in **Table 2**. All 114 patients successfully underwent SPLT-TLDG (73, 64.0%) or LADG (41, 36.0%). None of the patients underwent open surgery. Intracorporeal anastomosis was successfully performed in all the patients in the SPLT-TLDG group. The mean operation time was similar in the SPLT-TLDG and LADG groups. In contrast, the mean intraoperative blood loss in the SPLT-TLDG group was significantly lower than that in the LADG group (P = 0.022). No significant differences were found in the number of LNs harvested, time to ambulation, time to first flatus, time to first liquid intake, length of postoperative hospital stay, and decreases in hemoglobin and albumin levels between the two groups. Nine patients (12.3%) in the SPLT-TLDG group had postoperative complications, which was not significantly different from the six patients (14.6%) in the LADG group (P = 0.777). One duodenal stump fistula, one anastomotic leakage, and two abdominal cavity infections occurred in the TLDG group, which recovered after treatment with peritoneal drainage and antibiotics. One patient in the TLDG group experienced an intra-abdominal hernia 17 days after the operation, which was cured by emergency surgery without intestinal resection. One gastrojejunal anastomotic stenosis occurred 1 month after surgery and was completely relieved after two endoscopic dilations. One patient in the LADG group experienced postoperative bleeding and recovered after treatment with hemostatic treatment and blood transfusion. In addition, anastomotic leakage, pancreatic fistula, and abdominal cavity infection occurred in some patients in the LADG group and were cured with peritoneal drainage and antibiotics. Other complications, including pulmonary infection, were also cured after a period of appropriate therapy.

**Table 2:** Comparison of surgical outcomes between SPLT-TLDG and LADG.

Characteristics	SPLT-TLDG(n = 73)	LADG(n = 41)	P value
Operation time (min)	176.2 ± 40.8	182.0 ± 40.7	0.467
Intraoperative blood loss (mL)	47.1 ± 34.3	77.1 ± 93.4	0.022
Harvested lymph nodes	23.7 ± 9.2	21.1 ± 8.6	0.075
Incision size (cm)	3.6 ± 0.6	7.1 ± 1.1	<0.001
Time to ambulation (days)	1.6 ± 0.8	2.2 ± 0.8	0.123
Time to first flatus (days)	2.5 ± 1.1	1.8 ± 0.9	0.265
Time to first liquid intake (days)	4.0 ± 1.8	4.2 ± 1.8	0.325
Postoperative hospital stay (days)	8.9 ± 4.4	9.5 ± 4.3	0.144
Decrease in hemoglobin (g/L)	11.5 ± 13.3	10.2 ± 6.7	0.876
Decrease in albumin (g/L)	8.0 ± 4.7	6.8 ± 3.8	0.122
Second operation	1	0	>0.999
Postoperative complications	9 (12.3%)	6 (14.6%)	0.777
Duodenal stump fistula	1	0	
Anastomotic leakage	1	1	
Anastomotic stenosis	1	0	
Pancreatic fistula	0	1	
Abdominal cavity infection	2	1	
Intra-abdominal hernia	1	0	
Pulmonary infection	1	1	
Pleural effusion	2	1	
Postoperative bleeding	0	1	
Wound infection	0	0	
Death	0	0	

Variables are expressed as mean ± standard deviation or as n (%).

SPLT, self-pulling and latter transected; TLDG, total laparoscopic distal gastrectomy; LADG, laparoscopy-assisted distal gastrectomy.

During the follow-up period of 6 months at least, none of the patients complained of reflux symptoms or experienced tumor recurrence or metastasis.

## Discussion

BI reconstruction, also called delta-shaped anastomosis, was first reported in 2002 (10) and was modified by Huang et al. to improve its safety and reliability (19). Although widely accepted, however, BI reconstruction could not be conducted if the remnant stomach was small or if the duodenal stump was short. RY reconstruction had a reduced risk and lower degree of residual gastritis and bile reflex than encountered with BI and BII reconstructions (14, 15, 20). Furthermore, RY reconstruction could expand the indications of TLDG, irrespective of whether the remnant stomach was small. However, RY is a complex process and has therefore not gained widespread acceptance in TLDG (14, 15). To simplify RY, we first used SPLT to complete RY reconstruction in TLDG and then evaluated the safety and feasibility of this novel method by comparing it with conventional RY in LADG. Compared with LADG, SPLT-RY in TLDG involved less intraoperative blood loss and had a similar operation time. This result was similar to that in other studies (21, 22). During the LADG, the remnant stomach was pulled out *via* a small invasion and anastomosis was performed in a relatively narrow operative field, which may cause more tissue trauma. However, in TLDG, we had a better visual field, and more accurate operation could be performed, especially on patients with obesity; this explains the decreased intraoperative blood loss in TLDG. The overall complication rate in SPLT-TLDG was as controllable as that in LADG, and no conversion to open surgery or death occurred in SPLT-TLDG; therefore, we believe that SPLT-RY is a safe and feasible reconstruction method in terms of short-term surgical outcomes.

**Figure 4:**
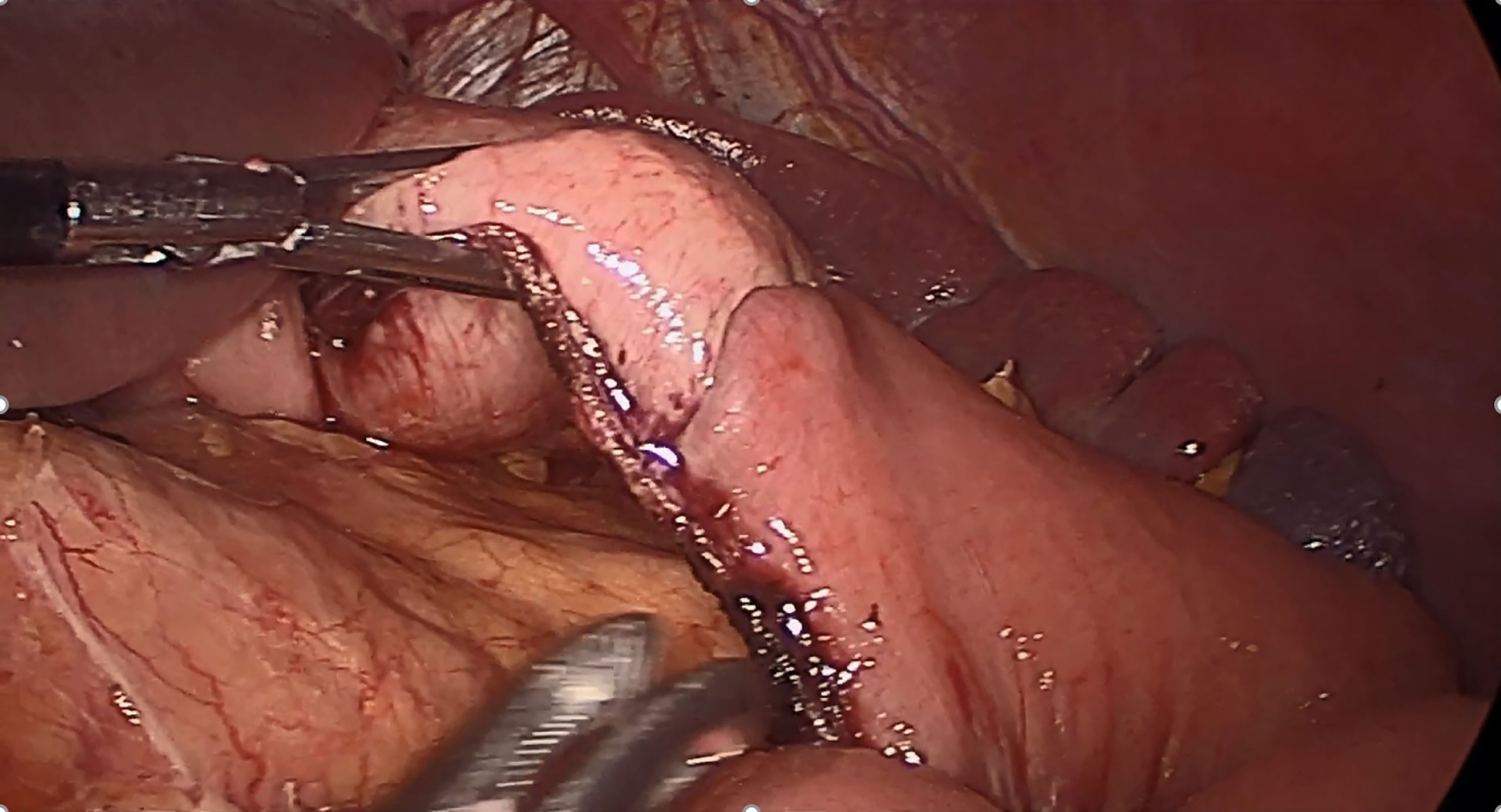
In SPLT-RY, we closed the common opening of the stomach and jejunum and cut off the stomach and jejunum in the same step, so that the stumps of the stomach and jejunum were in a completely straight line. SPLT-RY, self-pulling, and later transected Roux-en-Y.

In contrast to conventional RY-TLDG, when performing RY reconstruction in TLDG, we used later transection techniques (16), and it required fewer staplers to close the common opening of the proximal stomach and distal jejunum. We cut off the stomach and jejunum by using a linear cutting stapler. Thus, SPLT-TLDG may reduce cost and simplify the reconstruction procedure. Furthermore, the stumps of the stomach and jejunum were in a straight line in SPLT-RY (**Figure 4**), which reduced the size of the cutting edge intersection, as compared with conventional RY, which might improve anastomosis security and diminished the tough hand suturing required to close the common opening. Moreover, when applying SPLT, the distal stomach was initially retained uncut so it could be used to draw the stomach to a proper position for reconstruction, which can reduce the difficulty of the procedure. However, when frozen sections during surgery are indispensable for determining the proximal margin, SPLT is not recommended, because the specimen cannot be obtained until the anastomosis is complete.

Gastrectomy is an onco-metabolic surgery, and diabetes and hypertension could be resolved after gastrectomy (23, 24). RY reconstruction and the extent of gastrectomy might contribute to the remission of diabetes and hypertension (23, 25, 26). In this study, SPLT-RY reconstruction was performed, and the extent of gastrectomy could be controlled using SPLT-TLDG. SPLT-TLDG might provide promising benefits to patients with concurrent gastric cancer and metabolic diseases.

There are several tips for applying SPLT-RY to TLDG. First, we performed SPLT-TLDG on the patient’s right side. During the SPLT-RY reconstruction procedure, all anastomoses were performed by the surgeon, except for closing the common opening of the proximal stomach and distal jejunum and cutting off the proximal stomach by means of a linear cutting stapler by the assistant through the left 12-mm trocar hole. The surgeon and assistant did not exchange their positions, thereby reducing anastomosis time. However, this requires more experienced skills in the assistant. We have also attempted to complete all anastomoses by the surgeon on the right side, but when closing the common opening of the proximal stomach and distal jejunum, and when cutting off the proximal stomach by means of a linear cutting stapler, the anastomosis was clearly twisted. Thus, having the assistant perform this procedure could resolve the problem. Second, punching a small hole in the small intestine through which the linear cutting stapler can enter minimizes damage to the small intestine. Furthermore, the common opening should be carefully checked to prevent bleeding from the intestinal cavity after anastomosis. Third, the transverse incision is made above the pubic symphysis so that it may be better concealed, particularly in patients with high esthetic demands.

This study had some limitations. First, this was a retrospective study, with limited data included. Second, this study aimed to evaluate the effectiveness and safety of SPLT-RY in TLDG. However, the follow-up time was relatively short, and its long-term outcomes were uncertain. A longer follow-up would be needed in future. Third, given the aim of this study, it could be better to compare it with conventional RY in TLDG; however, because there were only approximately 20 patients who underwent conventional RY-TLDG in our center and as all these procedures were performed before implementation of SPLT-TLDG at our institution, considering the experience level according to the period, we did not have enough comparable conventional RY-TLDG samples in our center. Fourth, although the data from our center showed that SPLT-TLDG was feasible, it does not address the learning curve for surgeons elsewhere.

## Conclusion

We introduced the SPLT-RY method in TLDG. Our study showed that SPLT-RY reconstruction in TLDG is a safe and feasible surgical method in terms of short-term surgical outcomes and that it can simplify reconstruction after gastric cancer surgery. A well-designed prospective study should be conducted in the future to validate the clinical efficacy of this reconstruction method.

## Data availability statement

The raw data supporting the conclusions of this article will be made available by the authors, without undue reservation.

## Ethics statement

The studies involving human participants were reviewed and approved by Medical Ethics Committee of Chongqing Medical University. Written informed consent for participation was not required for this study in accordance with the national legislation and the institutional requirements.

## Author contributions

DC contributed to the conception and design of the study. KQ provided study materials and patients. FY and CT collected and assembled data. DC wrote the first draft of the manuscript. SW contributed to manuscript modification. All authors contributed to manuscript revision and read and approved the submitted version.

## Funding

This study received funding from the Chongqing Medical Scientific Research Project (Joint project of Chongqing Health Commission and Science and Technology Bureau) General Program under Grant number 2021MSXM096. The funder was not involved in the study design, collection, analysis, interpretation of data, writing of this article, or decision to submit it for publication.

## Acknowledgments

The authors are grateful to all their colleagues who helped in the preparation of this article.

## Conflict of interest

The authors declare that the research was conducted in the absence of any commercial or financial relationships that could be construed as potential a conflict of interest.

## Publisher’s note

All claims expressed in this article are solely those of the authors and do not necessarily represent those of their affiliated organizations, or those of the publisher, the editors and the reviewers. Any product that may be evaluated in this article, or claim that may be made by its manufacturer, is not guaranteed or endorsed by the publisher.
